# Reciprocal Regulation of KCC2 Trafficking and Synaptic Activity

**DOI:** 10.3389/fncel.2019.00048

**Published:** 2019-02-20

**Authors:** Etienne Côme, Martin Heubl, Eric J. Schwartz, Jean Christophe Poncer, Sabine Lévi

**Affiliations:** ^1^INSERM UMR-S 1270, Paris, France; ^2^Sorbonne Université, Paris, France; ^3^Institut du Fer à Moulin, Paris, France

**Keywords:** GABA_A_R, chloride homeostasis, membrane turnover, lateral diffusion, clustering

## Abstract

The main inhibitory neurotransmitter receptors in the adult central nervous system (CNS) are type A γ-aminobutyric acid receptors (GABA_A_Rs) and glycine receptors (GlyRs). Synaptic responses mediated by GlyR and GABA_A_R display a hyperpolarizing shift during development. This shift relies mainly on the developmental up-regulation of the K^+^-Cl^−^ co-transporter KCC2 responsible for the extrusion of Cl^−^. In mature neurons, altered KCC2 function—mainly through increased endocytosis—leads to the re-emergence of depolarizing GABAergic and glycinergic signaling, which promotes hyperexcitability and pathological activities. Identifying signaling pathways and molecular partners that control KCC2 surface stability thus represents a key step in the development of novel therapeutic strategies. Here, we present our current knowledge on the cellular and molecular mechanisms governing the plasma membrane turnover rate of the transporter under resting conditions and in response to synaptic activity. We also discuss the notion that KCC2 lateral diffusion is one of the first parameters modulating the transporter membrane stability, allowing for rapid adaptation of Cl^−^ transport to changes in neuronal activity.

## Introduction

**Figure 1:**
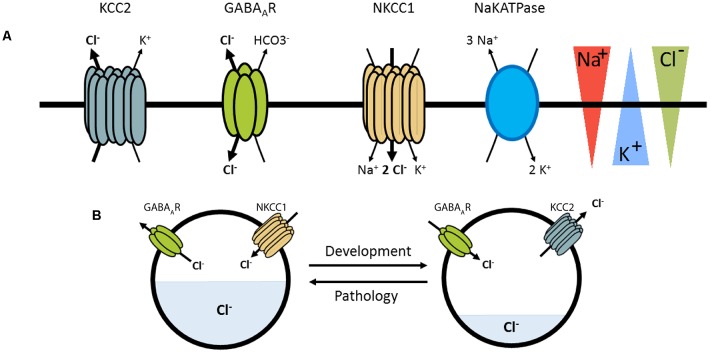
Pivotal role of KCC2 and NKCC1 in the regulation of [Cl]_i_.** (A)** KCC2 and NKCC1 transport chloride across the plasma membrane according to the Na^+^ and K^+^ gradients imposed by the Na^+^K^+^ ATPase. γ-aminobutyric acid receptor (GABA_A_R) are primarily permeable to chloride ions and thus GABAergic transmission depends on KCC2 and NKCC1 regulation. **(B)** Depending on pathological conditions or developmental stages, GABAergic transmission may be depolarizing, correlated with an increased NKCC1/KCC2 functional ratio.

Excitatory and inhibitory neurotransmission depend on the electrochemical ion gradients across the plasma membrane. The activation of postsynaptic ionotropic glutamate receptors leads to an influx of positively charged ions and thereby generates a depolarizing, excitatory postsynaptic potential (EPSP). In contrast, the net effect of activation of ionotropic anion permeable channels, such as type A γ-aminobutyric acid receptors (GABA_A_Rs) or glycine receptors (GlyRs), depends on the gradient of anions across the plasma membrane, predominantly chloride (Cl^−^) and bicarbonate (HCO_3_^−^; Bormann et al., 1987; Kaila and Voipio, 1987). The chloride gradient is mainly established by two secondary active transporters: the K^+^-Cl^−^ cotransporter KCC2 that extrudes chloride out of the neuron using the potassium gradient (generated by the Na^+^/K^+^ ATPase), and the Na^+^-K^+^-Cl^−^ cotransporter NKCC1 which usually transports chloride into the neuron based on transmembrane sodium and potassium gradients also generated by the Na^+^/K^+^ ATPase (Figure 1). Hence, the balance of expression and activity of these transporters influence intracellular chloride concentration ([Cl^−^]_i_) and the efficacy and polarity of GABAergic and glycinergic transmission. In immature neurons, where NKCC1 expression predominates, high [Cl^−^]_i_ is associated with depolarizing responses to GABA and glycine reflecting Cl^−^ efflux. In contrast, an increased expression of KCC2 in mature neurons lowers [Cl^−^]_i_ leading to an influx of Cl^−^ ions and hyperpolarizing responses upon GABA_A_R/GlyR activation.

In addition to its role in maintaining low [Cl^−^]_i_, KCC2 regulates the formation (Li et al., 2007), functional maintenance and plasticity (Gauvain et al., 2011; Fiumelli et al., 2013; Chevy et al., 2015; Llano et al., 2015) of glutamatergic synapses. Consistent with its key role in regulating inhibitory and excitatory neurotransmission, alterations in KCC2 expression and function have emerged as a common mechanism underlying pathological activity in a variety of neurological and psychiatric disorders (Medina et al., 2014; Kahle and Delpire, 2016; Moore et al., 2017; Wang et al., 2018). Understanding the mechanisms regulating KCC2 expression and function is therefore crucial to develop novel and efficient therapeutic strategies. Here, we will review the cellular and molecular mechanisms controlling KCC2 turnover and describe how these mechanisms are rapidly tuned when neuronal activity is challenged.

## KCC2 Structure and Regulatory Sequences

**Figure 2:**
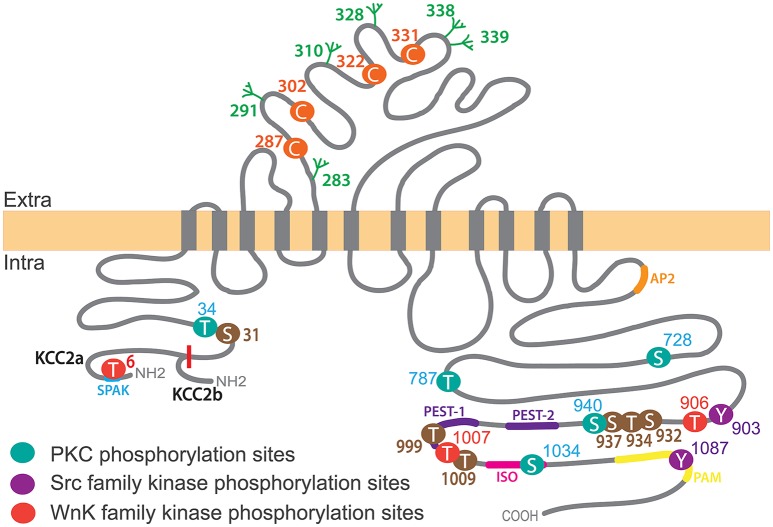
KCC2 structure, key phosphorylation residues and regulatory domains. The KCC2 co-transporter is a ~140 kDa protein with a predicted topology of 12 membrane spanning segments, an extracellular domain between transmembrane domains 5 and 6 containing N-glycosylation sites (green) and key Cysteine residues for ion transport (orange), and is flanked by two cytoplasmic carboxy- and amino- terminal domains. All residues (besides Threonine T6 which is only present in KCC2a) are numbered according to human KCC2b. Note that the mouse sequence lacks amino acid 1,000 and thus beyond this residue the numbering is shifted (e.g., T1007 in human corresponds to T1006 in mice). KCC2a and KCC2b isoforms differ from their intracellular amino-terminal domain with KCC2a having a 23 amino acids longer sequence bearing a SPAK-binding domain (blue). Other regulatory sequences of KCC2 are located within the large intracellular carboxy-terminal domain (CTD), such as protein kinase C (PKC) phosphorylation sites (Serine S728, T787, S940 and S1034), Src family kinase phosphorylation sites (Tyrosine Y903, Y1087) and WNK-SPAK-OSR1 kinases (T906, T1007). Other regulatory sequences are present on this region: the ISO domain (pink) allowing for KCC2 activity under isotonic conditions, and protein associated with Myc (PAM; yellow), Proline/E/S/T (PEST; purple), SPAK (blue) and adaptor protein-2 (AP2; orange) binding sequences. Note that KCC2 activators staurosporine and N-ethylmaleimide (NEM) act by controlling (de)phosphorylation of series of residues (brown).

KCC2 is one of nine members of the cation-chloride co-transporter (CCC) family encoded by the genes *Slc12a1-9*. KCC2 is a glycoprotein of 120 kDa with a predicted structure of 12 transmembrane segments (TMs), six extracellular loops flanked by a short intracellular amino terminal domain (NTD; amino acids 1–103) and a long intracellular carboxy-terminal domain (CTD; last 500 amino acids; Hartmann and Nothwang, 2015; Figure 2).

Two different KCC2 isoforms, KCC2a and KCC2b, are produced by use of alternative promoters of the *Slc12a5* gene encoding KCC2 (Uvarov et al., 2007). The NTD of KCC2a is 23 amino acids longer than the KCC2b one (Uvarov et al., 2007) and contains a putative SPAK (STE20/SPS1-related, proline alanine-rich kinase) and OSR1 kinase (Oxydative stress response 1) interaction site (de Los Heros et al., 2014; Table 1). Both isoforms show similar ion transport properties when expressed in human embryonic kidney (HEK) 293 cells and cultured hippocampal and cortical neurons (Uvarov et al., 2007; Markkanen et al., 2017), but have different subcellular localization *in vivo* (in neurons of the deep cerebellar nucleus, the pons and the medulla) and *in vitro* (cultured hippocampal neurons; Markkanen et al., 2014, 2017), suggesting a contribution of the NTD to the subcellular targeting of the transporter in given cells, probably *via* the binding to selective partners.

**Table 1:** Key regulatory sites and sequences on KCC2.

Site	Localization	References
Protein Associated with Myc (PAM)	1069-1088	Garbarini and Delpire (2008)
LVLLNMPGPPRNRNGDENYM		
PEST sequence	PEST-1: 949-966	Mercado et al. (2006)
	PEST-2: 974-1002	
ISO domain	1021-1035	Mercado et al. (2006)
PSPVSSEGIKDFFSM		
AP-2 interaction domain	657-662	Zhao et al. (2008)
LLRLEE		
SPAK-OSR1 interaction domain		
RFTV	4–7	Piechotta et al. (2002)
Cysteines	287-302-322-331	Hartmann et al. (2010)
N-Glycosylation sites	283-291-310-328-338-339	Agez et al. (2017)
Tyrosines	34-787-906-1007	Rinehart et al. (2009), de Los Heros et al. (2014), Weber et al. (2014) and Cordshagen et al. (2018)
	34, 999, 1009	
	34, 1009	
Serines	728-940-1,034	Lee et al. (2007), Weber et al. (2014) and Cordshagen et al. (2018)
	31, 913, 932, 988	
	25, 26, 937, 1022, 1025, 1026	

*Positions are shown relative to the human KCC2b protein*.

Based on a study of KCC1 (Casula et al., 2001), the KCC2 NTD has been suggested to be mandatory for KCC2 function (Li et al., 2007). Several groups have therefore used KCC2 lacking the NTD (KCC2-ΔNTD) to study ion-transport independent roles of KCC2 (Li et al., 2007; Horn et al., 2010; Fiumelli et al., 2013). The group of Igor Medina recently described altered exocytosis by truncation of the NTD in N2a cells, HEK 293 cells and cultured hippocampal neurons (Friedel et al., 2017).

In addition to five short extracellular loops, KCC2 contains a long extracellular loop (LEL) between TM5 and TM6 of around 100 amino acids (Williams et al., 1999). Based on KCC4 studies, N-linked glycosylation in the KCC2 LEL was proposed to be crucial for the membrane targeting of KCC2 (Hartmann and Nothwang, 2015). Six glycosylation sites on KCC2 were subsequently identified (Agez et al., 2017). Three human KCC2 mutations associated with severe early-onset epileptic encephalopathy showed reduced protein glycosylation and cell surface KCC2 (Stödberg et al., 2015), implicating KCC2 glycosylation in the control of its membrane expression. In addition, four highly conserved cysteine residues within the LEL were shown to be important for KCC2 activity but not membrane expression in HEK 293 cells probably due to their implication in inter- or intra-molecular di-sulfide bonds and correct protein folding (Hartmann et al., 2010).

The KCC2 CTD contains most of the KCC2 regulatory sequences. Complete truncation of the CTD reduces membrane expression of KCC1, 2 and 3 in *Xenopus laevis* oocytes and HEK 293 cells (Payne, 1997; Casula et al., 2001; Howard et al., 2002). Using live-cell surface labeling, Friedel et al. (2017) recently showed in cultured hippocampal neurons that KCC2 CTD is dispensable for membrane delivery of the transporter but is required for its membrane stabilization. Consistent with these observations, truncation of the KCC2 CTD by the Ca^2+^-dependent protease calpain at an unknown site leads to the internalization and lysosomal degradation of KCC2 in rat brain slices (Puskarjov et al., 2012). Moreover, the interaction of KCC2 CTD with the clathrin-binding adaptor protein-2 (AP-2) *via* a di-leucine motif induces a constitutive, dynamin-dependent and clathrin-mediated endocytosis of KCC2 in HEK 293 cells (Zhao et al., 2008). The CTD also hosts the majority of KCC2 phosphorylation residues (Figure 2) which influence KCC2 membrane stability and thereby function through regulation of the transporter’s lateral diffusion, oligomerization, clustering, and endocytosis (see below).

In contrast to other KCCs, KCC2 is constitutively active under isotonic conditions (Payne, 1997). A short sequence called ISO domain (1,022–1,037) located in the CTD has been shown to be responsible for this specific feature in Xenopus oocytes and hippocampal neurons (Mercado et al., 2006; Acton et al., 2012). Thus, replacement of this sequence by the corresponding KCC4 amino acids abolished constitutive KCC2 activity (Acton et al., 2012). Interestingly, KCC2 transporters lacking the ISO domain can still be activated under hypotonic conditions, indicating that two distinct domains are involved in KCC2 activation under isotonic vs. hypotonic conditions.

## Temporal and Spatial Expression Pattern of KCC2

KCC2 expression can be observed throughout the central nervous system (CNS) including spinal cord (Hübner et al., 2001), thalamus (Barthó et al., 2004), cerebellum (Williams et al., 1999), hippocampus (Rivera et al., 1999), cortical structures (Gulyás et al., 2001) and the auditory brainstem (Blaesse et al., 2006). Although KCC2 expression is very broad in the CNS, the reversal potential of GABA_A_R-mediated currents (E_GABA_) varies among neuronal populations and brain structures (Chavas and Marty, 2003; Watanabe and Fukuda, 2015). These differences are thought to reflect changes in CCC expression and function.

### Developmental Expression

Developmental upregulation of KCC2 expression has been described in different systems including human (Dzhala et al., 2005; Sedmak et al., 2016), mouse (Hübner et al., 2001), rat (Gulyás et al., 2001), zebrafish (Zhang et al., 2010), *C. elegans* (Tanis et al., 2009) and other species (for review Blaesse et al., 2009; Kaila et al., 2014). The KCC2 expression profile is well correlated with the sequential maturation of different brain regions (Watanabe and Fukuda, 2015), and follows the rostro-caudal axis of neuronal maturation (Li et al., 2002; Stein et al., 2004). Interestingly only the KCC2b isoform is developmentally upregulated, while KCC2a expression remains constant over brain maturation (Yeo et al., 2009). In the neonatal mouse brainstem KCC2a therefore contributes to about 20%–50% of the total KCC2 mRNA expression, while in the mature cortex its contribution decreases down to 5%–10% (Uvarov et al., 2009). KCC2a is expressed in the basal forebrain, hypothalamus and spinal cord, but is absent from the hippocampus (Markkanen et al., 2014). In contrast to full KCC2 knockout mice, which die at birth due to respiratory failure (Hübner et al., 2001), KCC2b knockout mice are viable until postnatal age 15 (P15; Woo et al., 2002). This suggests that both KCC2a and KCC2b isoforms are essential but contribute differentially to brain development and the establishment of inhibitory neurotransmission. Indeed, Dubois et al. (2018) recently showed a transient role of KCC2a at birth controlling the pontine neuromodulation of the respiratory motor circuits.

### Subcellular Expression

At the cellular level, KCC2 expression can be found in the somatodendritic plasma membrane in most brain regions, such as cerebellum (Williams et al., 1999), hippocampus (Rivera et al., 1999; Gulyás et al., 2001) or cortex (Szabadics et al., 2006). KCC2 membrane expression is enriched near inhibitory and excitatory synapses and in spine heads of hippocampal neurons (Gulyás et al., 2001; Hübner et al., 2001; Blaesse et al., 2006; Gauvain et al., 2011; Chamma et al., 2012). At the presynaptic level, only developing photoreceptor cells (Zhang et al., 2006) and retinal bipolar cells (Vardi et al., 2000) exhibit KCC2 expression. Axonal exclusion of KCC2 from CNS axons, including axon initial segment (Williams et al., 1999; Hübner et al., 2001; Chamma et al., 2012), leads to higher [Cl^−^]_i_ in axons than in the somatodendritic compartment (Price and Trussell, 2006). As a consequence, activation of GABA_A_R by GABA spillover or axo-axonic GABAergic synapses leads to increased axonal excitability (Stell et al., 2007; Ruiz et al., 2010; Pugh and Jahr, 2011, 2013; Stell, 2011).

Association of KCC2 with the plasma membrane increases during neuronal maturation. Hence, immature neurons show brighter intracellular labeling than mature neurons (Gulyás et al., 2001; Szabadics et al., 2006) and KCC2 forms clusters at the surface of mature neurons (Gulyás et al., 2001; Hübner et al., 2001; Barthó et al., 2004; Watanabe et al., 2009; Chamma et al., 2012, 2013; Heubl et al., 2017). In primary cultures of hippocampal neurons, KCC2 protein expression can be observed already at 3 days *in vitro* (div) in the soma, while the somatodendritic labeling peaks only at div 15 (Ludwig et al., 2003).

Markkanen and colleagues were the first to compare the subcellular distribution of the two KCC2 isoforms, KCC2a and KCC2b, in the deep cerebellar nucleus, the pons and the medulla, in hippocampal cultured neurons (Markkanen et al., 2014, 2017). The authors showed that in these neurons, KCC2a and KCC2b only partly colocalize and that the two isoforms are not localized in the same subcellular compartments in mature neurons (with stronger labeling of KCC2b on the soma and plasma membrane in general). The functional consequence of this distinct isoform localization however remains unclear.

## Molecular and Cellular Mechanisms of Regulation of KCC2

KCC2 is regulated at the transcriptional and post-transcriptional level (e.g., through phosphorylation/dephosphorylation of key residues) which in turn influence its cellular trafficking (cell surface delivery, membrane diffusion-trapping, clustering, surface removal and intracellular degradation).

### Transcriptional Regulation of KCC2

The neuron-specific KCC2 expression pattern is tightly regulated by transcription factors and neuron-restrictive silencing elements (NRSE) in the KCC2 gene *Slc12a5* (Karadsheh and Delpire, 2001; Uvarov et al., 2005, 2006; Yeo et al., 2009). Two NRSE sequences were found in intron 1 of the *Slc12a5* gene (Karadsheh and Delpire, 2001) and in the upstream regulatory region (Yeo et al., 2009). Binding of each of the restrictive elements to a neuron-restrictive silencing factor/repressor-element transcription factor (NRSF/REST) is sufficient to repress gene transcription (Yeo et al., 2009). In addition to these negative regulatory elements, two positive regulatory regions in the *Slc12a5* gene have been reported. Binding of the neuron specific transcription factor Egr4 (early growth response 4) to the Egr (early growth response) binding site activates KCC2 transcription (Uvarov et al., 2006). Similarly, Markkanen et al. (2008) found that binding of upstream stimulation factors, USF1 and 2, to an enhancer box (E-box) activates KCC2 expression.

The brain-derived neurotrophic factor (BDNF) has been shown to modulate KCC2 expression (Poo, 2001; Rivera et al., 2002; Aguado et al., 2003; Gottmann et al., 2009; Watanabe and Fukuda, 2015). While BDNF promotes KCC2 expression in immature neurons, exposure of mature CA1 pyramidal neurons to BDNF leads to decreased chloride extrusion (Rivera et al., 2004). Conversely, GABA increases BDNF expression in immature hippocampal and cerebrocortical neurons but not in mature hippocampal neurons, indicating a synergistic effect of GABAergic maturation and BDNF (Berninger et al., 1995; Kuczewski et al., 2011; Porcher et al., 2011). However, BDNF depletion (as shown in BDNF knockout mice) does not affect the developmental upregulation of KCC2 expression and function (Puskarjov et al., 2015). These results contrast with the reduced hippocampal KCC2 expression observed in TrkB knockout mice (Carmona et al., 2006) and the BDNF-induced increase in KCC2 mRNA expression in immature hippocampal neurons (Aguado et al., 2003; Rivera et al., 2004; Ludwig et al., 2011). Altogether, these results support a role of BDNF and TrkB in the developmental upregulation of KCC2.

Other trophic factors such as insulin-like growth factor 1 (Kelsch et al., 2001) and neurturin (Ludwig et al., 2011) have been implicated in the regulation of KCC2 expression. These data indicate that several signals control KCC2 expression and interact to increase KCC2 expression during neuronal development. The correlation of synaptic maturation with KCC2 upregulation therefore suggests their reciprocal influence.

### Posttranslational Regulatory Mechanisms

Ion-transport activity of KCC2 does not only depend on KCC2 expression levels but also on the abundance and activity of numerous other proteins such as scaffolding proteins, cytoskeleton interactors/regulators, kinases and phosphatases that regulate its cellular trafficking.

#### Exocytosis

Consistent with the developmental switch of GABA/glycine neurotransmission, translocation of KCC2 from the cytoplasm to the plasma membrane indicates that exocytosis contributes to the control of KCC2-mediated chloride extrusion. Dynamic visualization of membrane insertion or internalization using recombinant proteins linked to pH-sensitive fluorophores helped to determine exocytosis-endocytosis trafficking of several neurotransmitter receptors (Petrini et al., 2014; Zhang et al., 2015). Since the NTD and CTD of KCC2 are both cytosolic, a pH-sensitive pHluorin tag was inserted in the second or third extracellular loop of the transporter (Friedel et al., 2015, 2017). Insertion of the tag loop of the transporter did not perturb the function of the protein and therefore this construct constitutes a useful tool to study KCC2 trafficking (Friedel et al., 2017). The expression of pHluorin-tagged KCC2 mutants with deletions of the N terminal (ΔNTD) or C terminal (ΔCTD) domain and the use of live-cell surface immunolabeling of heterologous cells or cultured hippocampal neurons revealed that the NTD is essential for KCC2 plasma membrane delivery whereas the CTD is critical to its membrane stability (Friedel et al., 2017).

Recently, insights into the regulatory mechanisms of KCC2 exocytosis were obtained as transforming growth factor β2 (TGF-β2) was shown to mediate translocation of KCC2 from intracellular pools to the plasma membrane in developing and mature hippocampal neurons (Roussa et al., 2016). The mechanism for TGF-β2-mediated KCC2 membrane translocation involves the Ras-associated binding protein 11b (Rab11b). KCC2-Rab11b interaction was recently confirmed in a native KCC2 interactome study (Mahadevan et al., 2017).

#### Oligomerization

Multimeric assembly has been demonstrated for a large number of members of the CCC family (Moore-Hoon and Turner, 2000; Casula et al., 2001, 2009; Starremans et al., 2003; Blaesse et al., 2006; Simard et al., 2007; Warmuth et al., 2009). KCC2 was shown to form KCC2a and KCC2b homo-dimers, as well as KCC2a-KCC2b, KCC2-KCC4 and KCC2-NKCC1 hetero-dimers, in biochemical assays from neuronal and heterologous cell lysates (Blaesse et al., 2006; Simard et al., 2007; Uvarov et al., 2009).

There are discrepancies in the literature regarding the proportions of KCC2 monomers, dimers and higher-order oligomers in neurons. Blaesse et al. (2006) showed that an increase of KCC2 oligomers parallels transporter activation in the developing brainstem (between P2 and P30) whereas Uvarov et al. (2009) found oligomerization already at P2 in various brain regions. Mahadevan et al. (2014, 2017) using native PAGE reported that KCC2 form monomers, dimers as well as higher molecular mass complexes. However, using similar approaches, Agez et al. (2017) detected KCC2 monomers and dimers but not higher-order oligomers. These discrepancies may arise from differences in both experimental assays (native perfluorooctanoate-PAGE vs. 3%–8% Tris-acetate NuPAGE; Blaesse et al., 2006; Uvarov et al., 2009) as well as detergents used for sample preparation (CALX-R3 vs. C12E9; Agez et al., 2017; Mahadevan et al., 2017). These limitations also apply to SDS-PAGE studies, as differences in sample preparation influence the proportion of KCC2 dimer-like complexes (Medina et al., 2014). In conclusion, it is not possible to compare the relative abundance of KCC2 monomers, dimers and higher-order oligomers between studies.

The oligomerization domain has not been identified to date. However, several studies showed self-assembling capability for the CTD of NKCC1 and an Archean CCC (Simard et al., 2004; Warmuth et al., 2009) and decreased oligomerization of KCCs truncated on the C-terminus, or mutated on tyrosine residue 1087 (Simard et al., 2007; Watanabe et al., 2009). This observation suggests that KCC2 CTD might be involved in the assembly of the transporters as observed in Xenopus oocytes and hippocampal cultures (Simard et al., 2007; Watanabe et al., 2009). Whether the monomeric KCC2 is active remains unclear. Several studies reported a correlation between decreased KCC2 oligomerization and reduced transport activity (Watanabe et al., 2009; Mahadevan et al., 2014). For instance, neuropilin and tolloid like-2 (Neto-2) assemble with the oligomeric forms of KCC2 and this interaction increases KCC2-mediated Cl^−^ extrusion in cultured hippocampal neurons (Ivakine et al., 2013). Similarly, the kainate receptor GluK2 subunit interacts with KCC2 and is critical to KCC2 oligomerization, surface expression and ion-transport function in hippocampal neurons (Mahadevan et al., 2014; Pressey et al., 2017). However, since changes in KCC2 oligomerization and surface expression occur in parallel, these observations do not demonstrate a causal link between KCC2 oligomerization and Cl^−^ transport.

#### Clustering

KCC2 forms clusters in the neuronal plasma membrane (Gulyás et al., 2001; Hübner et al., 2001; Barthó et al., 2004; Watanabe et al., 2009; Chamma et al., 2012, 2013; Heubl et al., 2017). Interestingly the majority of KCC2 clusters are found at excitatory and inhibitory synapses in hippocampal cultures, without preferential accumulation at one type of synapses (Chamma et al., 2013). Ultrastructural studies indicate that KCC2 accumulates at the periphery of synapses in dendritic spines as well as on the dendritic shaft (Gulyás et al., 2001; Báldi et al., 2010).

KCC2 clustering could help to localize and/or stabilize transporters in sub-membrane compartments (e.g., near excitatory and inhibitory synapses), and to form a barrier in dendritic spines surrounding glutamatergic postsynaptic densities. Moreover, KCC2 clustering has been proposed to regulate the cotransporter function. Watanabe et al. (2009) showed that inhibition of tyrosine phosphorylation or deletion of a nearby region (Δ1089–1116) both lead to disruption of KCC2 clustering and transport activity without any change in the neuronal membrane pool. This suggests that the KCC2 CTD is involved in cluster formation and that clustering and function of the transporter are tightly correlated. Overexpression of the CTD on the other hand causes a decrease in KCC2 cluster size with no alteration of cluster density or chloride transport in hippocampal neurons (Chamma et al., 2013), suggesting KCC2 clustering does not rely exclusively on its CTD binding to the cytoskeleton. Association of KCC2 with lipid rafts was proposed to influence KCC2 clustering. Watanabe et al. (2009) observed that association with lipid rafts increases KCC2 clustering and function in neuronal cultures, while Hartmann et al. (2009) found larger clusters and enhanced transport activity after disruption of lipid rafts. The later study, however, was performed in HEK 293 cells and showed an overall increase in KCC2 surface expression. It therefore remains unclear how clustering of KCC2 in lipid rafts modifies its transport activity in neurons.

#### Lateral Diffusion

Lateral diffusion is a key mechanism controlling rapid activity-dependent changes in neurotransmitter receptor number (and therefore clustering) at synapses, a phenomenon underlying the tuning of synaptic transmission and plasticity (Choquet and Triller, 2013). Receptors constantly alternate between periods of free Brownian-type motion outside synapses and constrained diffusion at synapses. They are captured and confined at synapses by transient interactions with postsynaptic scaffolding molecules that anchor them to the underlying cytoskeleton. A reduced density of scaffolding proteins at synapses and/or a weakening of receptor-scaffold interactions increases the escape of receptors from synapses and thereby clustering and synapse efficacy. Since KCC2 is similarly clustered near excitatory and inhibitory synapses, we addressed the role of lateral diffusion on KCC2 subcellular distribution and function. This was analyzed using Quantum-based Single Particle Tracking (QD-SPT) in hippocampal cultures (Chamma et al., 2012, 2013; Heubl et al., 2017).

These experiments showed that KCC2 displays free Brownian-type motion outside clusters while it is slowed down and confined within clusters located near excitatory and inhibitory synapses (Chamma et al., 2012, 2013). However, KCC2 escapes clusters faster near inhibitory synapses than excitatory synapses, reflecting stronger molecular constraints at excitatory synapses. Further investigations suggested specific tethering of KCC2 near excitatory synapses through actin-binding of the CTD of KCC2 *via* the actin binding protein 4.1 N, whereas KCC2 is confined at inhibitory synapses by a distinct mechanism (Chamma et al., 2013). Therefore, KCC2 undergoes a diffusion-trap mechanism similar to neurotransmitter receptors.

**Figure 3:**
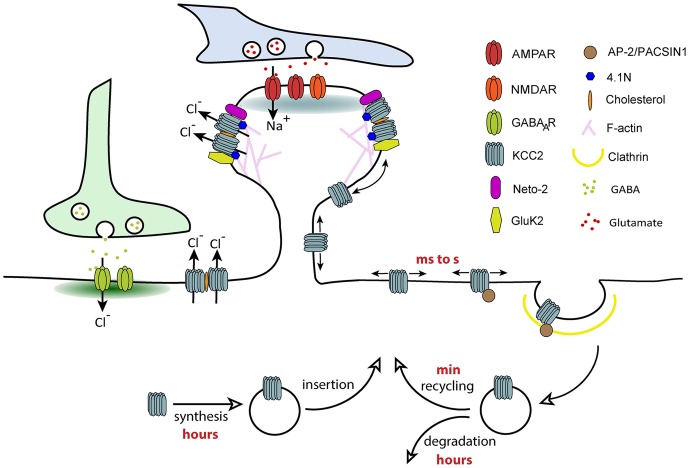
Regulation of KCC2 membrane trafficking by lateral diffusion, clustering and endocytosis. Different subpopulations of KCC2 exist in the plasma membrane: freely moving KCC2 outside of clusters and confined KCC2 in membrane clusters. KCC2 clustering probably results from its accumulation in lipid-rafts, interaction with the cytoskeleton *via* protein 4.1N and oligomerization of the transporter. Freely moving molecules are more susceptible to interact with molecules involved in clathrin-dependent endocytosis such as AP-2. Confinement of KCC2 in membrane clusters may therefore prevent transporter endocytosis, a mechanism favoring chloride extrusion. The balance between “freely moving” and “clustered” pools of KCC2 can be rapidly changed by activity through phosphoregulations, which regulate the overall density of transporters localized at the membrane.

KCC2 lateral diffusion is rapidly tuned by activity. Enhancing glutamatergic excitation or reducing GABAergic inhibition both increased KCC2 membrane diffusion (Chamma et al., 2013; Heubl et al., 2017) through reduced phosphorylation of S940 and increased phosphorylation of T906/1007, respectively. Changes in transporter diffusion were accompanied by cluster dispersion and increased membrane turnover of the transporter. Therefore, we propose that different subpopulations of transporters exist in the plasma membrane: freely moving KCC2 outside clusters and transporters confined in clusters in the vicinity of synapses. These two pools of transporters are in a dynamic equilibrium that can vary in response to changes in synaptic activity. The extra-cluster pool of transporters can be considered as a reserve pool in equilibrium with the perisynaptic pool. Transitions between these compartments by lateral diffusion may then participate in the fine tuning of synapses in response to local fluctuations of synaptic activity (Figure 3). Since changes in KCC2 mobility occur within tens of seconds (Heubl et al., 2017), lateral diffusion is probably the first cellular mechanism modulating the transporter membrane stability. This may represent a rapid mechanism for adapting Cl- homeostasis to changes in synaptic activity.

#### Endocytosis

While activity-dependent KCC2 endocytosis was shown to rapidly decrease its neuronal membrane pool (Lee et al., 2011; Chamma et al., 2013; Heubl et al., 2017), KCC2 turn-over rate under basal conditions is controversial. Two studies showed a high turn-over rate (of about 20 min) of the transporter in neuronal cultures (Lee et al., 2010) and rat hippocampal slices (Rivera et al., 2004). In contrast, Puskarjov et al. (2012) observed no change in KCC2 membrane pool in hippocampal slices after 4 h inhibition of protein synthesis (by cycloheximide) or degradation (by leupeptin). Although the authors concluded that KCC2 has a rather low turnover, what they were testing in this study was the lifetime of the transporter. Once KCC2 is synthesized and inserted in the membrane, it undergoes several cycles of endocytosis and exocytosis until final degradation. The lifetime of the transporter therefore seems to be >4 h whereas KCC2 turn-over rate at the membrane is in the range of 20–30 min.

Lee et al. (2010) reported increased surface expression of endogenous KCC2 in hippocampal cultured neurons after a 45 min exposure to dynasore, a cell-permeable inhibitor of dynamin. Using co-immunoprecipitation experiments, Zhao et al. (2008) showed that endogenous KCC2 interacts with the clathrin-binding AP2, suggesting that KCC2 internalization may be controlled by the clathrin-mediated endocytic pathway. Then, they identified in HEK293 cells a constitutive, non-canonical endocytic 657LLRLEE662 motif in the KCC2 CTD. Both di-leucine residues are required to mediate efficient transporter endocytosis but the L658 residue is the most important. The two glutamic acid residues downstream regulate the function of the di-leucine endocytic motif. This motif is highly conserved among KCC family members but not in NKCC1, NKCC2 or NCC proteins (Zhao et al., 2008). Furthermore, protein kinase C (PKC) and casein kinase substrate in neurons protein 1 (PACSIN1), which are involved in clathrin-mediated endocytosis and vesicle transport in neurons (Schael et al., 2013), were recently identified in a KCC2 interactome study (Mahadevan et al., 2017). Altogether these results suggest that KCC2 membrane retrieval may require AP2 and PACSIN1. PACSIN1 has been shown to regulate the activity-dependent AMPAR surface recycling in cerebellar neurons (Anggono et al., 2013; Widagdo et al., 2016). More work is now needed to test whether PACSIN1 plays a similar role in activity-dependent membrane recycling of KCC2.

However, regarding clathrin-dependence of KCC2 endocytosis, it is important to mention that the motif identified by Zhao et al. (2008) in an artificial overexpression system is non-canonical. Whether this motif plays a role in neurons and whether other regions on KCC2 are critical for internalization remains to be tested. Endocytosis of most transmembrane molecules involves post-translational modifications favoring interaction with the clathrin pathway that have not been clearly demonstrated for KCC2.

#### Degradation

Constitutively internalized transporters are not targeted for lysosomal degradation in HEK 293 cells (Zhao et al., 2008). Only upon increased glutamatergic activity does KCC2 undergo lysosomal degradation. This has been shown in spinal cord neurons following peripheral nerve injury (Zhou et al., 2012), in cultured hippocampal neurons and hippocampal slices upon application of the glutamate receptor agonist NMDA (Lee et al., 2011; Puskarjov et al., 2012) or interictal-like activity induced by Mg^2+^ depletion (Puskarjov et al., 2012). This process has been shown to require Ca^2+^-activated calpain cleavage of the KCC2 CTD (Puskarjov et al., 2012; Zhou et al., 2012). The exact location of the proteolytic cleavage site remains unknown. However, since it was proposed that KCC2 S940 dephosphorylation is a pre-requisite for calpain cleavage (Chamma et al., 2013), the calpain cleavage site may be positioned near the S940 residue.

#### Phospho-Regulation of KCC2

Phosphorylation or dephosphorylation of KCC2 key tyrosine, serine or threonine residues tune KCC2 activity mainly by controlling its membrane stability. KCC2 stability and clustering at the plasma membrane is directly regulated *via* its CTD and notably Y1087 and Y903 residues in HEK 293 cells, GT1-7 cells and hippocampal neurons (Watanabe et al., 2009; Lee et al., 2010). Other pathways have also been identified, and their consequences on KCC2 membrane stability and function characterized (Lee et al., 2007; Rinehart et al., 2009; Heubl et al., 2017). Thus, PKC-dependent phosphorylation of KCC2 S940 was shown to increase the transporter membrane stabilization in HEK 293 cells and in hippocampal neurons (Lee et al., 2007). Interestingly, S940 phosphorylation and calpain-mediated cleavage have been negatively correlated in cultured hippocampal neurons (Chamma et al., 2013). Studies in HEK 293 cells reported that T1007 phosphorylation is mediated by the serine/threonine kinase WNK1 [With No lysine (K) serine-threonine kinase 1] and its downstream effectors SPAK and OSR1 (Rinehart et al., 2009; de Los Heros et al., 2014). In contrast, T906 is not the target of WNK, SPAK or OSR1 in HEK 293 cells (de Los Heros et al., 2014; Zhang et al., 2016). The nature of the kinase phosphorylating T906 is still unknown (Zhang et al., 2016). The phosphorylation of T906 and T1007 keeps E_GABA_ depolarized by decreasing the membrane pool of KCC2 both in immature neurons (Friedel et al., 2015) as well as in mature neurons in response to reduced GABA_A_R activation (Heubl et al., 2017).

Other KCC2 phospho-sites have been identified in large-scale phospho-proteomics studies: S31, T34, S913, S932, S988, T999, T1009 (according to human sequence; Cordshagen et al., 2018), and S25, S26, T34, S937, T1009, S1022, S1025 and S1026 (Weber et al., 2014). Several of these sites tune KCC2 transport activity but unlike Y1087, S940, T906 and T1007, this regulation does not involve changes in total or surface expression levels of the transporter. Phosphorylation of S932, T934, S937 and dephosphorylation of T1009 enhance KCC2 transport function in HEK 293 cells (Weber et al., 2014; Cordshagen et al., 2018). Moreover, two potent KCC2 activators, N-Ethylmaleimide (NEM) and staurosporine, differentially impact KCC2 transport activity through a complex mechanism of (de)phosphorylation of several of these phospho-sites (Weber et al., 2014; Conway et al., 2017; Cordshagen et al., 2018). Staurosporine triggers phosphorylation of S932 and dephosphorylation of T1009. The action of staurosporine on T1009 occurs indirectly by inhibiting a kinase while its effect on S932 would be due to an indirect inhibition of a phosphatase (Cordshagen et al., 2018). NEM increases the phosphorylation of S940 while it decreases the phosphorylation of T1007 (Conway et al., 2017). NEM is thought to dephosphorylate T1007 through the control of SPAK phosphorylation/activity (Conway et al., 2017). Furthermore, a complex regulatory mechanism of KCC2 activity by staurosporine and NEM likely involves a change in the transporter conformational state through the (de)phosphorylation of several, partly overlapping phospho-sites that include S31, T34 and T999 for staurosporine and S31, T34 and S932 for NEM (Cordshagen et al., 2018). The function of other phosphorylation sites (e.g., S25, S26, S1022, S1025 and S1026), however, remains unclear.

## Regulation of KCC2 Cellular Trafficking by Neuronal Activity

KCC2 mRNA, protein, and surface expression are known to be down-regulated under pathological conditions such as epilepsy or in experimental paradigms leading to enhanced excitatory activity, including long term potentiation (LTP; Wang et al., 2006a), rebound burst activity (Wang et al., 2006b), repetitive postsynaptic spiking activity (Fiumelli et al., 2005), coincident pre- and post- synaptic spiking (Woodin et al., 2003), NMDAR activation (Kitamura et al., 2008; Lee et al., 2011), and epileptiform activity (Reid et al., 2001; Rivera et al., 2004; Pathak et al., 2007; Li et al., 2008; Shimizu-Okabe et al., 2011). Most of these paradigms result in a depolarizing shift in E_GABA_ due to a reduced KCC2 function and/or expression. Recently, KCC2 down-regulation was also observed in conditions of reduced GABAergic inhibition in mature neurons (Heubl et al., 2017). This raises questions about the cellular and molecular mechanisms controlling KCC2 activity. A mechanism has emerged that involves phospho-regulation of key KCC2 serine and threonine residues that in turn influence the membrane dynamics, clustering, endocytosis, recycling and/or degradation of the transporter (Lee et al., 2011; Puskarjov et al., 2012; Zhou et al., 2012; Chamma et al., 2013; Heubl et al., 2017).

### KCC2 Downregulation by Neuronal Excitation

Under conditions of increased neuronal activity, KCC2 diffusion is rapidly increased leading to the dispersal of KCC2 clusters, transporter internalization, degradation and ultimately deficits in chloride transport (Lee et al., 2011; Puskarjov et al., 2012; Zhou et al., 2012; Chamma et al., 2013). These effects are mediated by NMDAR-dependent Ca^2+^ influx, Ca^2+^-induced protein phosphatase 1 (PP1) dephosphorylation of KCC2 S940 and Ca^2+^-activated calpain protease cleavage of KCC2 CTD. These data suggest that deficits in KCC2 activity induced by dephosphorylation of S940 may contribute to the development of status epilepticus *in vivo*. However, the importance of KCC2 S940 phospho-regulation *in vivo* remains unclear. KCC2 dephospho-mimetic S940 (S940A) knock-in mice display normal basal KCC2 expression levels and activity in the hippocampus and do not exhibit any overt behavioral abnormality. Only in conditions of hyperactivity, S940 mice showed increased lethality to kainate-induced seizures (Silayeva et al., 2015). It remains to be determined whether increased lethality reflects enhanced seizure severity due to altered chloride transport or a brainstem-mediated respiratory arrest. On the other hand, phosphorylation of T906/1007 inhibits KCC2 function (Rinehart et al., 2009). Mice in which T906/1007 phospho-dependent inactivation was prevented by mutation into alanine showed increased KCC2 transport function in basal conditions (Moore et al., 2018). This effect was not associated with increased KCC2 surface expression but seems to involve changes in the intrinsic properties of the transporter. Importantly, increased KCC2 function attenuates chemically-induced epileptiform activity in T906A/1007A mice, both in acute hippocampal slices and *in vivo* (Moore et al., 2018), suggesting that enhancing KCC2 activity through T906/1007 dephosphorylation may be an effective approach in epilepsy treatment.

#### Implication in Glutamatergic Long-Term Potentiation

LTP of glutamatergic synapses in cortical neurons relies mainly on NMDAR activation and Ca^2+^-dependent activation of intracellular kinases such as Ca^2+^/calmodulin-dependent protein kinase II (CaMKII; Poncer, 2003). Consistent with the Ca^2+^-dependent regulation of the transporter, persistent (>1 h) KCC2 downregulation has been reported during hippocampal LTP (Wang et al., 2006a). Reduced KCC2 function was then hypothesized to dampen GABAergic transmission and to promote LTP at excitatory synapses (e.g., Ferando et al., 2016), although this hypothesis has not been tested experimentally. Instead, chronic KCC2 knockdown by RNA interference was shown to preclude LTP expression in hippocampal neurons (Chevy et al., 2015). This effect was independent of Ca^2+^ and CaMKII activation but instead involved the direct interaction of KCC2 with the Rac1-specific guanilyl exchange factor betaPIX. Loss of this interaction upon KCC2 suppression led to enhanced activation of Rac1 and its downstream effectors PAK1 and LIM kinase, which inhibit the actin-severing protein cofilin (Chevy et al., 2015; Llano et al., 2015). Thus, KCC2 suppression prevented actin depolymerization required for activity-driven AMPAR exocytosis during LTP (Gu et al., 2010). This effect however was observed upon chronic KCC2 knockdown. How acute downregulation of KCC2 upon physiologically induced LTP influences subsequent plasticity therefore remains to be tested.

### Regulation of KCC2 by GABAergic Inhibition

Several studies have shown that KCC2 can be rapidly downregulated by enhanced neuronal activity and glutamatergic neurotransmission (see above). However, little was known until recently about the regulation of KCC2 by synaptic inhibition. A study by Woodin et al. (2003) reported that increased GABAergic transmission also leads to KCC2 downregulation. However, this study was carried out in immature neurons displaying mainly depolarizing excitatory GABA_A_R-mediating synaptic responses associated with activation of voltage-dependent Ca^2+^ channels (VDCCs) and intracellular Ca^2+^ signaling pathways (Woodin et al., 2003). Therefore, this study did not reveal regulation of KCC2 by synaptic inhibition *per se* but instead by excitatory GABAergic transmission.

**Figure 4:**
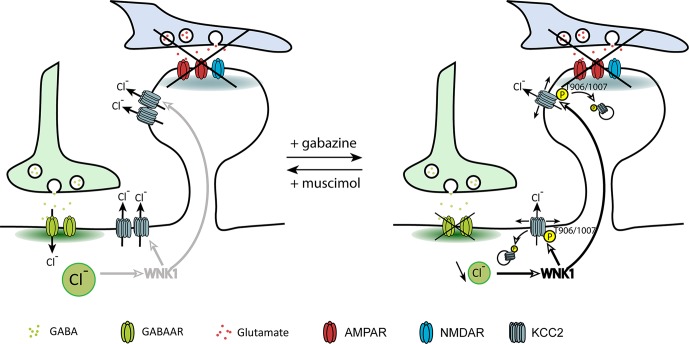
Regulation of KCC2 diffusion and membrane stability by GABAergic inhibition. The impact of inhibitory activity on KCC2 diffusion was explored while blocking neuronal activity and glutamate receptors activity with the Na^+^ channel blocker tetrodotoxin, the ionotropic glutamate receptor antagonist kynurenic acid, and the group I/group II mGluR antagonist R, S-MCPG. Chloride influx through GABA_A_Rs inhibits WNK1, leading to KCC2 dephosphorylation on threonines 906/1007 and membrane stabilization. Upon GABA_A_R blockade, reduced intracellular chloride activates WNK1 leading to KCC2 phosphorylation on threonines 906/1007 and increased KCC2 diffusion and subsequent internalization. From Heubl et al. (2017).

A recent study from our group investigated the direct contribution of GABAergic inhibition in the regulation of KCC2 and chloride homeostasis in mature neurons (Heubl et al., 2017). In order to isolate the effect of GABAergic inhibition on KCC2 diffusion and membrane stability, we adjusted neuronal inhibition while blocking action potentials and glutamate receptors. In these conditions, increasing neuronal GABA_A_R-mediated synaptic inhibition with muscimol enhanced KCC2 diffusion constraints and membrane stability. On the other hand, GABA_A_R activity blockade with gabazine increased KCC2 diffusion while reducing its membrane clustering and stability (Figure 4). Although these observations reflect the influence of bath application of GABA_A_R agonists and antagonists on KCC2 membrane diffusion and stability, one could imagine that such regulation may also take place locally near GABAergic synapses.

**Figure 5:**
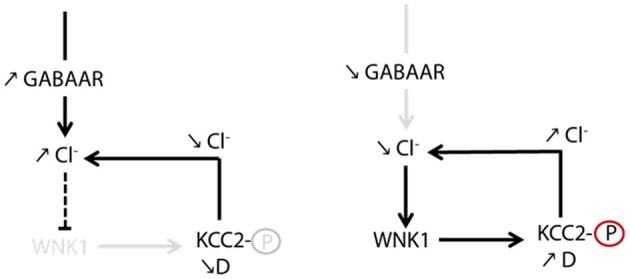
Homeostatic regulation of intracellular chloride *via* WNK1-dependent KCC2 phosphorylation. GABA_A_R-dependent chloride influx inhibits WNK1 kinase activity and stabilizes KCC2 in the membrane. Increased KCC2-dependent chloride extrusion can therefore counteract efficiently chloride influx due to activation of GABAergic synapses. In conditions of decreased GABA_A_R activation, a decrease in the intracellular chloride level activates WNK1. Subsequent increase in KCC2 diffusion (D) and loss of KCC2 in the membrane contribute to restore intracellular chloride concentration. From Heubl et al. (2017).

The search for the signaling pathway underlying the GABA_A_R-dependent regulation of KCC2 demonstrated for the first time that Cl^−^ acts as a genuine second intracellular messenger to rapidly tune inhibitory synaptic transmission (Heubl et al., 2017). Thus, lowering intracellular Cl^−^ levels activates the Cl^−^-sensing WNK1 kinase which in turn phosphorylates and activates the SPAK and OSR1 kinases. Activated SPAK and OSR1 phosphorylate KCC2 T1007, leading to decreased KCC2 activity (Heubl et al., 2017). Conversely, increasing [Cl^−^]_i_ *via* photostimulation of halorhodopsin eNpHR, the light-activated microbial chloride pump, significantly reduced diffusion coefficients and increased the confinement of KCC2 transporters. This mechanism would therefore allow neurons to locally increase or decrease their KCC2 pools to match GABAergic synaptic activity and subsequent need to extrude Cl^−^ (Figure 5). We concluded that GABAergic inhibition in mature neurons tunes itself *via* rapid regulation of KCC2-mediated changes in intracellular Cl^−^ levels. Since the effect of eNpHR was observed 10 s after light exposure, diffusion-trap of KCC2 appears to be a very rapid mechanism to control Cl^−^ homeostasis in neurons.

However, the published values of the Cl^−^ sensitivity of WNK1 measured in an *in vitro* kinase assay (Piala et al., 2014) cannot account for the activation of this signaling pathway upon GABA_A_R activity changes in neurons (Heubl et al., 2017). Indeed, intracellular chloride levels in these cells are expected to be in the range of 5–10 mM under control conditions, down to 4–6 mM after GABA_A_R blockade and up to 10–15 mM upon GABA_A_R activation. The IC50 of chloride was about 20 mM for WNK1 autophosphorylation (Piala et al., 2014) and 100 mM for phosphorylation of its target SPAK in an *in vitro* kinase assay (Terker et al., 2016). If transposable *in situ*, these values would suggest WNK1 would be constantly activated in neurons. Instead, we showed modulation of WNK1 activity even upon subtle intracellular chloride changes (Heubl et al., 2017). The chloride sensitivity of WNK1 in neurons remains to be determined. Additional mechanisms such as membrane translocation (Zagórska et al., 2007) or interaction with other ion-sensitive molecules could modulate the chloride sensitivity of WNK1 upon GABA_A_R blockade. Further characterization of WNK1 localization and activity in neurons would therefore provide invaluable insights into how changes in osmolarity and ion concentration can tune its kinase activity.

This signaling pathway may also participate in the onset of pathological conditions. Indeed, a single subcutaneous injection of the GABA_A_R antagonist pentylenetetrazole (PTZ) to elicit epileptic seizures *in vivo* increased WNK1, SPAK and OSR1 phosphorylation/activities and promoted KCC2 T906/1007 phosphorylation, which resulted in KCC2 inhibition in neuronal cells (Heubl et al., 2017). Interestingly, PTZ injection also increased NKCC1 T203/T207/T212 phosphorylation. Considering threonine phosphorylation was shown to have opposite effects on KCC2 vs. NKCC1 activity (McCormick and Ellison, 2011), the downregulation of the WNK/SPAK/OSR1 pathway could be a very efficient mechanism to adjust neuronal Cl^−^ homeostasis in disorders associated with altered inhibition like epilepsy, schizophrenia, autism and neuropathic pain.

Since KCC2 S940 residue is crucial for the regulation of KCC2 transport activity (see above), one may wonder about the interplay between KCC2 T906/1007 and S940. KCC2 diffusion was increased upon gabazine application even when S940 was mutated to aspartate, indicating that threonine phosphorylation can destabilize KCC2 in the membrane independently of its S940 phospho-status (Heubl et al., 2017). On the other hand, NMDAR-dependent S940 dephosphorylation in neurons in which KCC2 T906/1007 were mutated into alanine also destabilizes KCC2 independently of the threonine phosphorylation status. Hence, the kinase pathways involved in KCC2 regulation by GABAergic inhibition and neuronal excitation appear to be largely independent.

#### Impact at Glutamatergic Synapses

**Figure 6:**
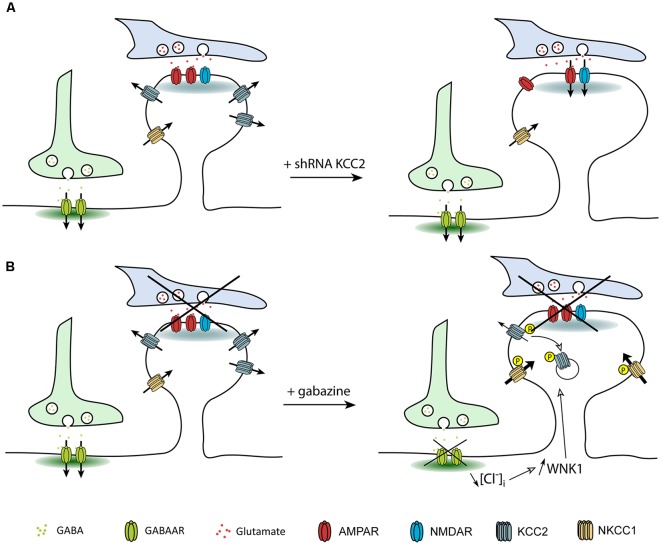
Regulation of dendritic spine head volume upon changes in KCC2 membrane expression. Arrows indicate water fluxes associated with the activity of secondary active transporters and ionotropic receptors. **(A)** Suppression of KCC2 expression leads to increased spine head volume due to the loss of KCC2-associated water extrusion. **(B)** Activation of WNK1 upon GABA_A_R blockade leads to KCC2 and NKCC1 phosphorylation and subsequent decrease and increase in the membrane stability of KCC2 and NKCC1, respectively. Increased water influx associated with NKCC1 activity cannot be counteracted by KCC2, leading to spine head swelling.

Several studies showed that, in addition to its role in setting [Cl^−^]_i_ in mature neurons, KCC2 also influences spine head volume as well as the efficacy of glutamatergic neurotransmission (e.g., Gauvain et al., 2011; Chevy et al., 2015). KCC2-mediated spine head volume regulation may rely on water fluxes associated with ion transport, as demonstrated for KCC- (Zeuthen, 1991a,b) and NKCC-mediated transport in epithelial cells (Hamann et al., 2005). Instead, the effect of KCC2 on glutamatergic transmission was shown to depend on its interaction with actin-related proteins (Gauvain et al., 2011). Thirty minutes of GABA_A_R blockade with gabazine also induced dendritic spine swelling while GABA_A_R activation with muscimol had no effect on dendritic spines (Heubl et al., 2017). The effect of gabazine is reminiscent of what was observed upon chronic KCC2 knockdown or pharmacological blockade. Spine swelling upon KCC2 blockade may thus result from ion and water influx associated with ionotropic receptor activation. Under basal activity, the number of opened receptors is low in hippocampal cultures, with spontaneous EPSP frequency ranging 20–50 Hz. However, gabazine-induced spine swelling occurred on a much faster time scale than with ionotropic glutamate receptors blockade (Gauvain et al., 2011; Heubl et al., 2017). Another mechanism could therefore be at play in the gabazine effect. WNK kinases regulate KCC2 and NKCC1 in opposite directions. Activation of WNK1 in conditions of reduced neuronal inhibition possibly leads to KCC2 removal from the plasma membrane while in the meantime NKCC1 is stabilized at the membrane (Vitari et al., 2006; McCormick and Ellison, 2011). Therefore, spine head swelling observed upon gabazine application may primarily involve increased NKCC1 membrane stabilization and water influx (Zeuthen and Macaulay, 2012). NKCC1 being very efficient in mediating water influx, this might explain the rapidity of the gabazine effect on dendritic spines as compared to that observed upon KCC2 blockade only (Figure 6). It would be interesting to directly test NKCC1 involvement in spine swelling upon gabazine application, for instance using the NKCC1 blocker bumetanide.

KCC2 knockdown leads to actin reorganization in spine heads (Chevy et al., 2015). Thus, reduced KCC2 content at the plasma membrane potentially weakens the molecular barrier formed by KCC2 in dendritic spines. This could in turn increase AMPAR escape from spines and reduce the efficacy of glutamatergic synapses as shown upon KCC2 knockdown (Gauvain et al., 2011). Therefore, we predict that KCC2 membrane removal upon reduced GABA_A_R activity may act to homeostatically adjust GABAergic and glutamatergic synaptic transmission.

#### Implication in Energy Loss

The regulation of KCC2 may not only permit a rapid reaction to changes in [Cl^−^]_i_ but also preserve energy consumption. Thus, the loss of KCC2 in conditions of decreased GABA_A_R-dependent Cl^−^ influx would maintain membrane KCC2 at minimal levels required to keep E_GABA_ hyperpolarized. Indeed, for every Cl^−^ ion extruded by KCC2, the transporter uses the energy of the electrochemical gradient of one potassium ion. The Na^+^/K^+^ ATPase that generates the potassium transmembrane gradient is the main energy consumer in the brain (Buzsáki et al., 2007; Harris et al., 2012; de Lores Arnaiz and Ordieres, 2014). Even though the highest energetic cost of the Na^+^/K^+^-ATPase is used to restore transmembrane potential upon action potential discharge (Harris et al., 2012; Howarth et al., 2012), maintaining low [Cl^−^]_i_ is associated with high metabolic cost (Kaila et al., 2014). Kaila et al. (2014) raising the hypothesis that “the downregulation of KCC2 following neuronal trauma may be part of a general adaptive cellular response that facilitates neuronal survival by reducing the energetic costs that are needed to preserve low [Cl^−^]_i_”. Under physiological conditions, rapid redistribution of KCC2 in the membrane could allow neurons to save energy by keeping surface KCC2 molecules at the minimum required density.

In conclusion, normal and pathological excitatory and inhibitory activities rapidly tune KCC2 function at both inhibitory and excitatory synapses. This regulation occurs through phosphorylation-induced changes in KCC2 membrane diffusion, clustering, endocytosis, recycling or degradation. Although alterations in excitatory and inhibitory signaling might have similar effects on KCC2 cellular trafficking and function, the underlying molecular mechanisms are distinct and involve Ca^2+^ vs. Cl^−^ signaling cascades and key serine and threonine KCC2 residues. The recent discovery of activity-dependent regulation of KCC2 by the Cl^−^-dependent WNK/SPAK/OSR1 signaling pathway is of particular interest in the pathology since it controls simultaneously KCC2 and NKCC1 in opposite directions. Further investigation will tell whether targeting this signaling pathway efficiently restores chloride homeostasis and synaptic inhibition in epilepsy, neuropathic pain and various neuropsychiatric disorders.

## Author Contributions

SL supervised the writing of the review and corrected the manuscript. JP gave advice. EC and MH participated equally in the writing of the review and ES wrote sub-sections of the manuscript.

## Conflict of Interest Statement

The authors declare that the research was conducted in the absence of any commercial or financial relationships that could be construed as a potential conflict of interest.
